# Variability Survey at Different Genetic Markers as Effective Tools for the Management of the Endangered Breeds: The Case of the Sicilian Native Donkeys

**DOI:** 10.3390/ani16010090

**Published:** 2025-12-28

**Authors:** Morena Carlentini, Serena Tumino, Giorgio Chessari, Aurora Antoci, Andrea Criscione, Donata Marletta, Salvatore Mastrangelo, Salvatore Bordonaro

**Affiliations:** 1 Dipartimento di Agricoltura, Alimentazione e Ambiente, University of Catania, 95123 Catania, Italy; morena.carlentini@phd.unict.it (M.C.); serena.tumino@unict.it (S.T.); giorgio.chessari@unict.it (G.C.); aurora.antoci@phd.unict.it (A.A.); andrea.criscione@unict.it (A.C.); salvatore.bordonaro@unict.it (S.B.); 2 Dipartimento di Scienze Agrarie, Alimentari e Forestali, University of Palermo, 90129 Palermo, Italy; salvatore.mastrangelo@unipa.it

**Keywords:** endangered breeds, donkey, molecular markers, conservation program, mating schemes

## Abstract

Domestic donkeys (*Equus africanus asinus*) are an important livestock genetic resource that is currently considered at risk. The number of donkey breeds worldwide is declining due to their loss of function as working animals in rural communities. Local breeds with small populations, which are threatened by genetic erosion and are at risk of extinction, require urgent action to characterize and preserve their genetic diversity. As microarrays containing thousands of single-nucleotide polymorphisms (SNPs) are not yet available for these species, traditional molecular markers such as microsatellites and mitochondrial DNA (mtDNA) remain valuable tools for genetic monitoring and management. This study uses different molecular markers to assess and manage genetic variability in endangered Sicilian donkey breeds. This information can support breeding plans and mating schemes, as well as in situ and ex situ conservation programs. The practical application of molecular information in the conservation strategies for these breeds is briefly discussed, and the proposed approach is considered transferable to other threatened breeds.

## Simple Summary

The mechanization of agriculture has led to a sharp decline in the number of donkey breeds worldwide. However, Italy has a long tradition of donkey breeding, and Sicily still preserves three native breeds, all of which are currently at risk of extinction. This study employed various molecular markers to assess and manage the genetic diversity of these endangered Sicilian donkey breeds for conservation programs. This approach can be transferred to other threatened breeds.

## 1. Introduction

Since its domestication, which occurred in North Africa about 6000 years ago [1], the donkey has been a faithful companion as a burden animal and a valuable aid in many agricultural activities. The mechanization of transport and agriculture has led to a strong reduction in the number of donkeys worldwide, especially in the developed countries. Despite renewed interest in donkey breeding for milk production [2,3,4] and for recreation and therapy purposes [5,6], donkey populations remain poorly managed and valorized [7].

The Mediterranean area represents the cradle of European donkey breeding [8], and in this context, Sicily holds a prominent position as it still preserves three native populations, namely, Ragusano, Pantesco, and Grigio Siciliano (Supplementary File S1). According to the FAO, these breeds are endangered, with a risk status ranging from “vulnerable” for Ragusano to “critical” for Pantesco and Grigio Siciliano. Since 2021, the National Association of Equine and Donkey Breeders (A.N.A.R.E.A.I.) has been responsible for managing the stud books of Italian horse and donkey breeds with limited diffusion, including the Ragusano and Pantesco. In contrast, the Grigio Siciliano population, also known as “Ferrante” due to its gray coat, has not yet been officially recognized as a breed. This small population is at risk of genetic erosion, which poses a threat to its biodiversity and could lead to extinction. To better characterize and safeguard these genetic resources, which are poorly characterized and managed, effective actions are needed to avoid further genetic diversity erosion.

Over the last few decades, microsatellite markers have been widely used in the analysis of inbreeding and genetic relationships in donkey populations [2,8,9,10]. These molecular markers are useful for developing more effective strategies to maintain healthy gene pools and preserve unique traits in native breeds. Analyzing genetic variability is, therefore, essential to ensure the conservation, sustainability, and resilience of livestock populations [11]. Although genomic analysis has recently made great strides in terms of both quality and cost, there is still a lack of high-throughput analysis tools for single-nucleotide polymorphisms (SNPs) in donkeys. Therefore, microsatellites or STR (short tandem repeats) remain among the most informative and widely used markers in donkeys, especially for the estimation of inbreeding level and the study of their evolution and biodiversity [12,13].

In addition to molecular markers of biparentally inherited nuclear DNA, mitochondrial DNA (mtDNA) markers are also used, mainly in phylogenetic studies focused on maternal inheritance [14]. The D-loop region is the most investigated and informative region of the mtDNA, and it has proved to be an ideal tool for tracing maternal lines, investigating the evolutionary history, phylogeny, and genetic structure of animal populations [15,16,17], for studying population migrations and interactions [18,19]. The combined use of autosomal and mitochondrial genetic markers for characterizing and managing small livestock populations is well established and has proved to be particularly useful in the *Equus* genus [12,20,21,22].

The aim of this research is the genetic characterization of the three native Sicilian donkey populations using both autosomal and uniparental molecular markers (microsatellites and mtDNA, respectively). We also discuss the practical application of these molecular data to the conservation programs of these breeds, along with an approach proposed as transferable to other threatened breeds.

## 2. Materials and Methods

### 2.1. Sampling and DNA Extraction

Blood samples were collected in K2-EDTA tubes from 272 donkeys belonging to three populations (Ragusano—RAG, 157; Pantesco—PAN, 57; and Grigio Siciliano—GRI, 58) reared on the island of Sicily (Southern Italy). A total of 66 males and 215 females with known pedigree and conforming to the morphological standards of the breed were sampled by using criteria of maximum representativeness of the population. All experimental procedures and sampling were approved by the Bioethics Committee of the University of Palermo: protocol code UNPA-CLE-98597. Blood samples were collected in compliance with the European rules (Council Regulation (EC) No. 1/2005 and Council Regulation (EC) No. 1099/2009) during routine health controls by the public veterinary service. The authors confirm that they have followed EU standards for the protection of animals used for scientific purposes.

The EZ1&2 DNA Blood 200 mL kit for the EZ2 Connect Qiagen instrument (Qiagen, Hilden, Germany) was used to extract genomic DNA.

### 2.2. Donkey Genetic Diversity Investigated by Microsatellite Markers (STRs)

All 272 individuals were characterized by outsourcing service using a set of 16 STR markers for individual identification and parentage testing; 13 STR (AHT04, ASB23, HMS02, HMS06, HMS07, HMS18, HMS3, HTG07, HTG10, TKY297, TKY312, TKY337, TKY343) are recommended by the International Society for Animal Genetics (ISAG) (https://www.isag.us/Docs/EquineGenParentage2021.pdf, accessed on 24 November 2025) while three are for internal laboratory use (AHT05, HTG06, and VHL20).

Molecular data were filtered for missing data, resulting in the elimination of the marker AHT05, which showed 38 individuals with missing genotypes (RAG, 30; PAN, 4; GRI, 4). Subsequently, the unbiased expected heterozygosity (He) [23], the observed heterozygosity (Ho), the mean number of alleles (MNA), the polymorphic information content (PIC) [24], the allelic richness (Ar) [25], the molecular inbreeding coefficient (F_IS_) [23], and the average differentiation index (F_ST_) were calculated.

Allele sharing distance [26] was estimated from genotypes matrix and visualized as a neighbor-joining tree using SplitsTree v.6.4.13 software [27].

To estimate the degree of genetic admixture among individuals from the three breeds, allele frequencies at the 15 STR loci were analyzed using STRUCTURE 2.3.4 software [28]. The admixture model associated with the correlated allele frequencies option [29] was implemented to infer the population’s structure using no prior information. The run length was set to 500,000 burn-ins followed by 500,000 iterations. The range of possible clusters (K) tested was between 1 and 10, and 10 different runs were performed for each K. The number of clusters that best fit our data was determined by plotting the mean ln Pr (X/K) over multiple independent runs for each K, as suggested by the authors.

### 2.3. Donkey Genetic Diversity Investigated by D-Loop Sequencing

A subsample of 81 donkeys (GRI—26, PAN—23, RAG—32) was selected according to the genealogical information in order to capture the majority of maternal genetic variability present in the three populations. The matrilinear diversity pattern was investigated by sequencing a portion of 479 bp of the D-Loop region of mtDNA [15]. Specific primers based on the reference sequence obtained from the NCBI database (GenBank NC_001788.1: https://www.ncbi.nlm.nih.gov/nuccore/NC_001788.1 accessed on 1 April 2024) were used to amplify the region included between nt 15386 and nt 15865, according to [15]. The PCR products were visualized by electrophoresis on 1.5% agarose gel stained with Gel Red (4 µL/80 mL), suddenly purified by digestion at 37 °C for 30 min with ExoSAP-IT, followed by enzymatic inactivation at 80 °C for 15 min, and, finally, sent for sequencing through an external service (BMR Genomics Srl, Padova—Italy). The chromatograms were visualized using Chromas software (Version 2.6.6). The sequences were aligned with ClustalW using MEGA v11.0 software [30]. The main descriptive statistics, such as the number of polymorphic sites (S), the number of haplotypes (NH), haplotype diversity (Hd), nucleotide diversity (π), and the average number of pairwise nucleotide differences (i.e., how much, on average, the sequences differ from each other), as well as the pairwise genetic differentiation (Kst), were calculated using the software DnaSP 6.12 [31].

Finally, a wide comparison was performed using a shorter sequence of 238 bp. A total of 200 genotypes from donkeys of different regions of the Mediterranean basin, the Balkans, Asia, and Africa were retrieved from the NCBI website (https://www.ncbi.nlm.nih.gov/ accessed on 1 April 2024) (Table 1). The genetic relationships among breeds were reconstructed by generating a distance matrix based on the simple number of nucleotide differences. The median-joining algorithm was applied to infer the network, which was visualized using PopArt v.1.7 software [32].

## 3. Results

### 3.1. Genetic Diversity, Relationships, and Admixture Analysis by Means of STRs

Only 14 microsatellites out of 15 were polymorphic across all the Sicilian donkey breeds, as HMS06 resulted as monomorphic in the Pantesco breed. The main statistics for each STR locus are presented in Table 2. In the entire set of Sicilian donkeys, the number of alleles per locus varied between 4 and 11. The mean Ar was 5.69. The polymorphism information content (PIC) ranged from 0.237 to 0.732 with an average value of 0.518. Overall, the observed heterozygosity (Ho) was slightly lower than the expected one (He). The diversity indices per breed showed moderate levels of genetic variability (Table 3).

The Pantesco donkey showed the lowest values of heterozygosity, mean number of alleles, and average polymorphic information content. Many private alleles were detected in Ragusano, whereas Grigio Siciliano showed the highest heterozygosity values. The molecular inbreeding coefficients, described by the F_IS_ fixation index, were lower than 0.05 in all the Sicilian populations.

**Figure 1:**
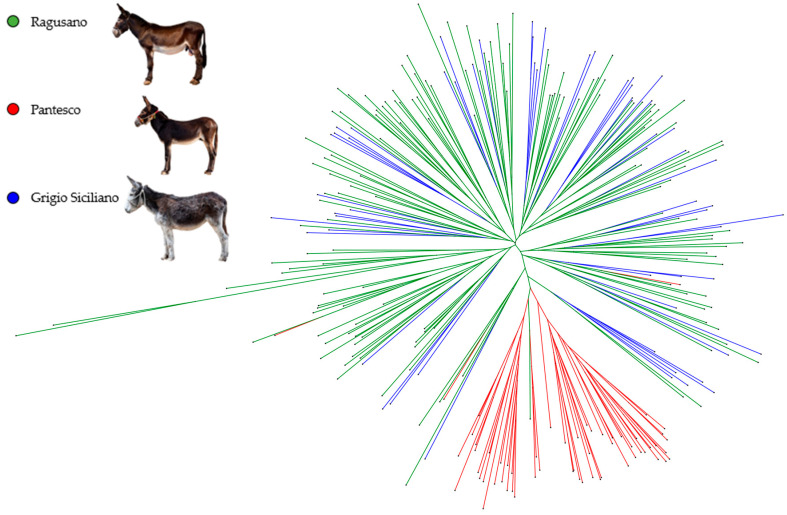
Neighbor-joining tree based on allele sharing distances (ASDs) of Ragusano, Pantesco, and Grigio Siciliano donkeys.

**Figure 2:**
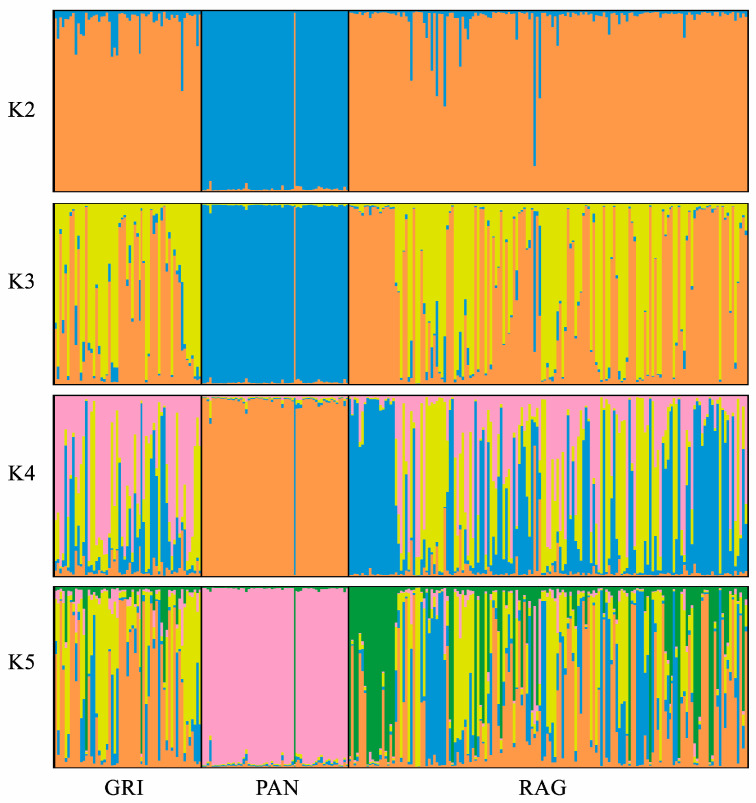
Linear graphs of the ancestral clusters (K) of the 3 Sicilian donkey populations, estimated by genomic structure analysis with STR markers.

The neighbor-joining phylogenetic tree based on the ASD matrix (Figure 1) highlights a clear distinction of the Pantesco donkeys from the rest of the analyzed sample. Most (95%) of the PAN donkeys derive from a single phylogenetic node and show a clear distinction from other populations. The exceptions are three individuals mixed with Grigio Siciliano and Ragusano, and one RAG donkey that clustered within the PAN group. In contrast, Ragusano and Grigio Siciliano displayed a very close relationship. Finally, a genetic structure analysis was conducted to estimate the degree of genetic admixture between individuals of the three genetic types, based on the assumption of a number of ancestral populations (K) ranging from 2 to 5 (Figure 2). The most probable number of clusters was found to be K = 2, clearly identifying the Pantesco donkey and the Ragusano–Grigio Siciliano grouping. As the number of ancestral groups increases, the Pantesco breed maintains its differentiation, while the Ragusano and Grigio Siciliano donkeys show shared genetic components, with under-structuring being particularly evident in the Ragusano breed.

### 3.2. Donkey Genetic Diversity Investigated by Uniparental, Maternal Markers (mtDNA)

The variability of the D-loop region sequenced in the three native Sicilian breeds is reported in Table 4. A total of 18 haplotypes were detected, with the highest diversity found in Grigio and Ragusano. Only two haplotypes were observed in Pantesco due to a G > A nucleotide in 15,821 and A > G nucleotide in 15,599. Haplotypes 5 and 4 were the most common in the Sicilian donkeys with frequencies of 0.40 and 0.17, respectively.

Maternal diversity among Sicilian donkeys was quite low, especially in Pantesco (Table 5). Nucleotide diversity (π) was very low in Pantesco (0.0008 ± 0.0003) and slightly lower in Grigio Siciliano (0.0220 ± 0.0030) than in Ragusano (0.0250 ± 0.0020). In contrast, Grigio Siciliano exhibited the highest haplotype diversity (Hd) at 0.871 ± 0.056, with a total of 13 haplotypes, of which 6 were private. Pantesco had only two haplotypes, both of which were shared with the other breeds. For pairwise genetic differentiation, Kst revealed significant differentiation between Pantesco and both Grigio (Kst = 0.133, *p* < 0.001) and Ragusano (Kst = 0.313, *p* < 0.001), whereas differentiation between Grigio and Ragusano was low but slightly significant (Kst = 0.049, *p* = 0.038) (Table S1).

**Figure 3:**
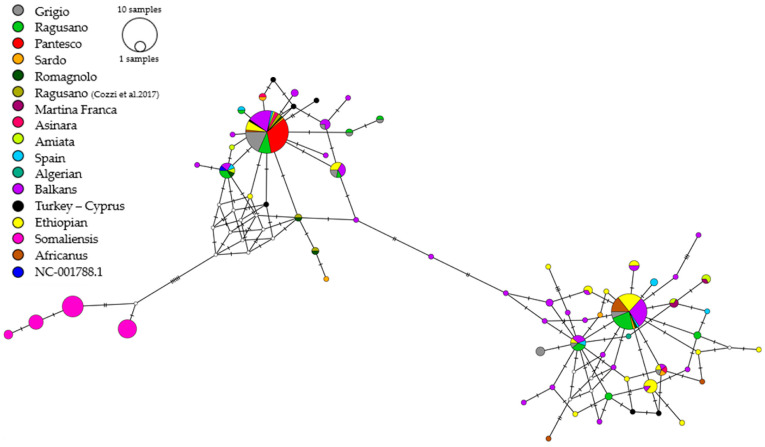
Haplotype network constructed from 281 sequences belonging to individuals of *Equus asinus*. Each circle represents a haplotype, and its size is proportional to its frequency. Lines indicate mutations between haplotypes. The colors correspond to the breeds according to the legend [18].

The median-joining (MJ) network constructed using 281 mtDNA D-loop sequences (238 bp) from Sicilian and other Mediterranean domestic donkeys, as well as Ethiopian, Somali, and Nubian wild asses, is presented in Table 1. Following the reduction in sequence length from 489 to 238, the number of Sicilian donkey haplotypes decreased from 18 to 13. The network distinctly identified three macro-haplogroups (Figure 3). Group 1 consists exclusively of Somali wild donkeys. Group 2 comprises many Italian breeds, including the majority of Sicilian donkeys (68%), all Pantesco donkeys, and several Grigio Siciliano donkeys which share haplotype H5. Group 3 comprises Ethiopian and Nubian wild donkeys, Balkan donkeys, and some Ragusano and Grigio Siciliano donkeys, primarily represented by haplotype H4. Overall, this analysis provided a detailed picture of maternal genetic diversity, revealing relevant differences among the Sicilian donkey breeds. 

## 4. Discussion

Next-generation sequencing (NGS) technologies enable high-throughput, cost-effective DNA analysis in major livestock genetics using microarrays containing tens to hundreds of thousands of single-nucleotide polymorphism (SNP) markers, but such a tool is currently unavailable for the donkey species. Consequently, although recent studies have employed NGS-based approaches, such as SNPs generated through ddRAD sequencing [40,41] and whole-genome sequencing (WGS) [42,43,44], traditional molecular markers, including microsatellites and mitochondrial DNA (mtDNA), remain economical, effective, and easy-to-interpret tools for the management and conservation of endangered donkey breeds. As demonstrated in several studies on *Equus* [12,20,21,22,35], the D-loop region of maternally ancestral mtDNA, together with microsatellite loci, provides robust evidence for assessing population genetic variation, evolutionary relationships, and the matrilineal origins of the species under investigation.

### 4.1. Genetic Diversity, Relationships, and Admixture Analysis by Means of STRs

The analysis of a set of autosomal markers has provided insights into biodiversity and the genetic relationship among Sicilian donkeys. Previous studies had investigated genetic variability at 15 and 16 STR loci in the three Sicilian donkey breeds [2,8]. Almost 15 years later, the genetic variability slightly increased in all three Sicilian native breeds. This result, based on a larger sample (272 vs. 108 and vs. 100 in Bordonaro et al. [2] and in Colli et al. [8], respectively) and with a partially different set of markers, is, nevertheless, encouraging. It confirms that the management and conservation activities carried out to date have preserved the genetic diversity of these threatened breeds. Accordingly, the molecular inbreeding coefficients, described by the F_IS_ fixation index values, were low. In Pantesco, the low level of variability as well as the absence of private alleles could be due to the severe bottleneck that occurred in the 1990s, when only a very small number of re-founders contributed to the “recovery” of this ancient breed. Despite the small number of effectives and the low internal variability, the low F_IS_ value indicates a careful mating policy. While this contradicts the ranking reported by Bordonaro et al. [2], it partly confirms other results by Colli et al. [8], which show a lower F_IS_ value in Pantesco than in Ragusano and other Italian breeds.

The neighbor-joining phylogenetic tree, summarizing the genetic relationships among individuals, confirmed the clear isolation of the Pantesco donkeys, which derive from a single phylogenetic node. The Pantesco breed exhibits a clear genetic distinctness, whereas Ragusano and Grigio Siciliano show a high degree of admixture and substructuring. This evidence was confirmed by the literature [2] and, more recently, by an interesting survey carried out on nine Italian donkey breeds, including the three Sicilian ones, using about 27K SNP markers generated by means of the double-digest restriction site associated DNA (ddRAD) sequencing technology [40]. Documented historical records also support this result. In fact, until 1950, stallions reared in Sicily had bay or gray coats, the coat colors of the current Ragusano and Grigio Siciliano, respectively, and morphological differentiation was based solely on body size relative to the breeding area [41] (p. 673).

The different nodes and branches present in the NJ tree allow us to visualize the genetic variability within the analyzed sample. The identification of genetic and family lines within Sicilian donkey populations is valuable for planning mating strategies aimed at increasing population size while preserving genetic variability and limiting the increase in inbreeding, which threatens livestock biodiversity in small populations.

### 4.2. Donkey Genetic Diversity Investigated by Uniparental, Maternal Markers (mtDNA)

Maternal inheritance plays a key role in the characterization and management of biodiversity in livestock populations and conservation programs [42,43,44]. The analysis of the D-loop region of mitochondrial DNA (479 bp) in a large sample of Sicilian donkey breeds provided a detailed picture of maternal genetic diversity, revealing differences among the three populations.

Ragusano and Grigio showed a rich maternal genetic variability, characterized by a substantial number of private haplotypes. As expected, very low variability, with no private haplotype, was recorded in the Pantesco breed, easily explained by its recent demographic history, when the breed has been reconstituted from a nucleus of six jennies [2]. The Ragusano breed displayed parameters higher than those reported by Cozzi et al. [18], who found a π value of 0.005 ± 0.0009 in a very limited sample of six animals. In contrast, they were lower than those reported by Mazzatenta et al. [19], who identified a nucleotide diversity (π) of 0.132 in a sample of 22 donkeys. These differences may be attributed to the number of animals, the sampling area and employed strategies, and the size of the analyzed D-Loop fragment. In fact, variation in the length of the sequenced stretch influences the number of polymorphic sites detected, subsequently affecting the genetic diversity parameters. Grigio Siciliano has very high levels of maternal genetic diversity in terms of haplotypic diversity (Hd = 0.871 ± 0.056) and nucleotide diversity (π = 0.022 ± 0.003) (Table 5). The number of private haplotypes is also high. These pieces of evidence confirm the value of the gray-coated donkey population as an important reservoir of biodiversity within the Sicilian donkey heritage.

The pairwise genetic differentiation based on Kst showed that Pantesco was differentiated from both Ragusano and Grigio, consistent with its reduced mitochondrial variability. Conversely, the low Kst between Grigio and Ragusano (Kst = 0.049; *p*-value = 0.038) suggests a closer matrilinear proximity.

In the MJ network, the Sicilian donkeys’ D-loop sequences were compared with those of Mediterranean and Balkan domestic donkeys as well as with African wild donkeys. This allowed the identification of two main clades, Clade 1 and Clade 2 (Figure 3), consistent with the known mitochondrial groups in the asinine species [1,15], recently renamed by Migliore et al. [16] as Haplogroup A and B, respectively. Most of the Sicilian donkeys, including the entire sample of Pantesco and the majority of Grigio Siciliano, belong to Clade 2, sharing haplotypes with Italian, Spanish, and Balkan breeds.

In this broader context, the Pantesco breed appears phylogenetically closer to *Equus africanus somaliensis*, a subspecies of the African wild ass (*Equus africanus*) native to Somalia and other regions of the Horn of Africa, and it is confirmed as the most genetically uniform with a single mitochondrial haplotype. This haplotype is also present in other Italian breeds, including Ragusano and Grigio, which is consistent with its history and the low number of effective breeding stock, which has resulted in a severe genetic bottleneck. These results support previous findings [19], indicating that the Pantesco is genetically distinct from the other Italian breeds and belongs to a single mitochondrial line.

In contrast, the distribution of the D-loop haplotypes observed in Ragusano and Grigio Siciliano individuals in two phylogenetic clades suggests a wide phylogenetic maternal inheritance. This geographic–genetic distribution indicates that Sicilian donkey populations derived from a complex set of genetic contributions, consistent with Sicily’s historical and geographical position in the Mediterranean basin. Ragusano donkeys are evenly distributed across both clades, whereas Grigio Siciliano donkeys appear more prevalent in Clade 2. Notably, Ragusano shares haplotypes with individuals from the Balkan and Mediterranean area, supporting the hypothesis of a historical connection with southeastern Europe, possibly linked to trade routes or to successive introductions of breeding stock over the centuries. This result, probably linked to the extensive dataset including 65 sequences belonging to many native breeds from nine different countries of the geographical area, aligns with findings reported by Cozzi et al. [18], who described a similar distribution in other Italian donkey breeds.

D-Loop sequencing allows the identification of various maternal lines in the Sicilian donkey breeds, an aspect that should be considered in mating plans to avoid the loss of rare haplotypes present in both in Ragusano and in Grigio Siciliano. The length of the analyzed sequences affects the amount of genetic information obtained, as longer sequences can highlight a greater number of polymorphic sites. This is evident in the Pantesco, where two haplotypes were identified when considering the long (489 bp) sequence, and only one when considering the short (238 bp) sequence.

### 4.3. Main Practical Implication in the Use of Molecular Information in the Management of the Endangered Sicilian Donkey Breeds

For the official recognition of a breed, a sufficient number of animals must undergo morphological and genetic characterization using molecular markers. In 2006, studies on Sicilian donkeys resulted in the official registration of the Pantesco breed. This ancient breed, considered extinct in the 1980s, was recovered starting from a pilot group of nine animals (six females and three males), selected based on morphotype and genetic analysis. Nowadays, there are 78 Pantesco donkeys in existence (38 jennies and 29 males) (https://www.anareai.it/le-razze-a-limitata-diffusione/asino-pantesco, accessed on 5 November 2025). Recently, as part of the recovery project, a small group of these donkeys was returned to Pantelleria, the island after which they are named.

In the last few years, the Grigio Siciliano population has been the subject of a demographic, morphological, and molecular study aimed at better defining its uniqueness and distinctiveness compared to other Sicilian donkey breeds (research project: “Genetic enhancement of Sicilian horse and donkey breeds”). Enrolment in the Stud Book of Equine Populations with Limited Distribution, managed by ANAREAI, would help to preserve the genetic heritage of the ancient Grigio Siciliano donkey. Gray-coated donkeys, ancestors of the current Grigio Siciliano, were historically abundant in Sicily. Until the middle of the last century, the stallions used in Sicilian breeding stations had either gray or bay coats and were both considered equally valuable. However, in 1953, the official definition of the Ragusano breed as exclusively bay in coat color excluded gray donkeys from selection and relegated them to a marginal role.

The analysis of autosomal molecular markers (STRs and SNPs) and mitochondrial markers (D-Loop) has confirmed historical interbreeding between the Ragusano and the Grigio Siciliano donkey breeds [2,8,40]. As part of the characterization and conservation project, the molecular information reported in this study was used to establish a pilot group of gray donkeys (16 jennies and 4 stallions) bred on four pilot farms in Sicily. Careful mating plans have already resulted in the birth of gray-coated foals with typical morphology of Grigio Siciliano. Furthermore, genetic characterization of the Sicilian donkeys will help regional institutions and the donkey breeders to plan rational mating schemes, ensuring the preservation of the endangered Ragusano and Pantesco breeds.

Finally, the conservation of threatened breeds must be supported by germplasm banks, which store and preserve precious genetic material, such as sperm, embryos, and oocytes, through cryopreservation techniques for future use. The molecular information presented here is currently being used to guide the collection of genetic materials in the ex situ germplasm conservation process for all three endangered Sicilian donkey breeds. To date, about 800 semen samples have been collected from seven stallions of the three Sicilian donkey breeds, which were selected based on their genetic and morphological characteristics.

## 5. Conclusions

In this survey, we employed microsatellites and mtDNA markers to monitor and manage Sicilian donkey populations. In a project aimed at characterizing and safeguarding donkey biodiversity, these markers have proven to be cost-effective and practical tools for identifying genetic lines and profiles of breeding animals with the aim of guiding mating schemes. The regular use of these tools for verification of the genealogies/pedigrees will enable the heritage of Sicilian donkey breeds to be better preserved and managed, and potentially selected, by monitoring genetic variability within and between breeds over time.

## Figures and Tables

**Table 1:** mtDNA sequences (238 bp long) used to construct the network.

Macro-Area	Code	Country	Sequences	References
Mediterranea and Balkan area	SIC	Italy	81	Present study
	ITY	Italy	28	[18]
	SPA	Spain	9	[33,34]
	BAL	Croatia, Albania, Bulgaria, Greece, Kosovo, Montenegro, Romania, Serbia, Ukraine	65	[35,36]
	TRK-CYR	Turkey, Cyprus	7	[37]
Africa	ETH	Ethiopia	39	[38]
	SOM	Ethiopia	41	[15,38,39]
	NUB	Ethiopia	10	[15,38,39]

**Table 2:** Summary statistics for 15 STRs in the Sicilian donkey breeds.

Locus	Ar	N	Ho	He	PIC
HTG10	8.6	10	0.657	0.752	0.714
VHL20	4.4	6	0.611	0.587	0.509
HTG07	10.4	11	0.789	0.769	0.732
AHT04	6.0	10	0.589	0.622	0.566
HMS3	5.6	7	0.460	0.504	0.448
HMS06	3.2	4	0.349	0.348	0.281
HMS07	6.2	8	0.257	0.253	0.237
HMS02	5.2	6	0.574	0.597	0.538
HTG06	4.2	5	0.570	0.585	0.527
ASB23	5.6	6	0.689	0.702	0.655
HMS18	7.3	10	0.565	0.645	0.577
TKY297	6.2	7	0.629	0.697	0.637
TKY312	4.6	6	0.551	0.601	0.522
TKY337	5.4	7	0.437	0.440	0.395
TKY343	3.5	4	0.562	0.534	0.427
**Mean**	**5.69**	**7.13**	**0.553**	**0.576**	**0.518**

Ar = allelic richness; N = number of alleles; Ho = observed heterozygosity; He = expected heterozygosity; PIC = polymorphic information content.

**Table 3:** Summary statistics for the Sicilian donkey breeds investigated by 15 STRs.

Breed	Size	MNA	Ar	Private	Ho ± SD	He ± SD	F_IS_ (95% C.I.)	Fst	PIC
GRI	58	5.27 ± 1.79	5.3	1	0.584 ± 0.017	0.617 ± 0.040 (−0.009–0.097)	0.044	0.041	0.559
PAN	57	3.00 ± 1.00	3.0	-	0.495 ± 0.017	0.498 ± 0.052 (−0.064–0.084)	0.010	0.081	0.435
RAG	157	7.07 ± 2.15	6.0	26	0.580 ± 0.010	0.612 ± 0.037 (0.020–0.079)	0.050	0.050	0.559

Size = population sample size; MNA = mean number of alleles; Ar = allelic richness; Private = number of private alleles; Ho = observed heterozygosity ± standard deviation; He = expected heterozygosity ± standard deviation; F_IS_ = average molecular inbreeding coefficient per microsatellite and population (95% confidence interval); Fst = average differentiation index of pairwise values between populations; PIC = polymorphic information content.

**Table 4:** Polymorphic sites and haplotypes of the mtDNA D-loop sequence (nt 15,386-15,865) in Sicilian donkeys.

**Haplotype**	**PAN**	**RAG**	**GRI**	**1** **5** **4** **8** **4**	**1** **5** **4** **9** **0**	**1** **5** **5** **0** **3**	**1** **5** **5** **2** **7**	**1** **5** **5** **3** **2**	**1** **5** **5** **6** **9**	**1** **5** **5** **8** **0**	**1** **5** **5** **9** **2**	**1** **5** **5** **9** **8**	**1** **5** **5** **9** **9**	**1** **5** **6** **2** **1**	**1** **5** **6** **2** **6**	**1** **5** **6** **4** **4**	**1** **5** **6** **4** **5**	**1** **5** **6** **5** **2**	**1** **5** **6** **6** **2**	**1** **5** **6** **9** **8**	**1** **5** **7** **1** **3**	**1** **5** **7** **1** **8**	**1** **5** **7** **4** **6**	**1** **5** **7** **4** **8**	**1** **5** **7** **7** **0**	**1** **5** **8** **0** **1**	**1** **5** **8** **0** **2**	**1** **5** **8** **0** **6**	**1** **5** **8** **2** **0**	**1** **5** **8** **2** **1**	**1** **5** **8** **2** **2**
NC_001788.1				G	C	T	C	A	A	A	A	C	A	A	A	G	A	C	A	C	C	C	G	G	T	C	T	C	C	G	G
H1		3		.	.	.	.	.	.	.	.	.	.	.	.	.	.	.	.	.	.	.	.	.	.	.	.	.	.	.	.
H2		3	1	A	T	C	.	.	G	G	.	T	G	.	.	A	.	T	G	T	.	.	.	.	C	T	.	T	T	A	A
H3			1	.	.	.	.	.	G	G	.	.	G	.	.	.	.	.	.	.	T	.	.	.	.	.	.	.	.	A	.
H4		11	3	A	T	C	.	.	G	G	.	T	G	G	.	A	.	T	G	T	.	.	.	.	C	T	.	T	T	A	A
H5	19	4	9	.	.	.	.	.	.	.	.	.	G	.	.	.	.	.	.	.	.	.	.	.	.	.	.	.	.	.	.
H6			1	.	.	.	.	.	.	.	.	.	G	.	.	.	.	.	.	.	.	.	.	A	.	.	.	.	.	.	.
H7			1	A	T	C	.	.	G	G	.	T	G	.	G	A	.	T	G	T	.	.	C	.	.	T	.	T	T	A	A
H8		1	2	.	.	.	.	.	.	G	.	.	G	.	.	.	.	.	.	.	.	.	.	.	.	.	.	.	.	.	.
H9			2	A	T	C	.	.	G	G	.	T	G	.	G	A	.	T	G	T	.	.	.	.	.	T	.	T	T	A	A
H10		1	1	.	.	.	G	.	.	.	.	.	G	.	.	.	.	.	.	.	.	.	.	.	.	.	.	.	.	.	.
H11			1	A	T	C	.	.	G	.	.	T	G	G	.	A	.	T	G	T	.	.	.	.	C	T	.	T	T	A	A
H12			2	.	.	.	.	.	.	.	.	.	G	.	.	.	.	.	.	.	.	.	.	.	.	.	C	.	.	.	.
H13		1	1	.	.	.	G	G	.	.	.	.	G	.	.	.	.	.	.	.	.	.	.	.	.	.	.	.	.	.	.
H14		1	1	.	.	.	.	.	.	.	.	.	G	.	.	.	.	.	.	.	.	T	.	.	.	.	.	.	.	.	.
H15	4	2		.	.	.	.	.	.	.	.	.	G	.	.	.	.	.	.	.	.	.	.	.	.	.	.	.	.	A	.
H16		2		A	T	C	.	.	G	G	G	T	G	G	.	A	.	T	G	T	.	.	.	.	C	T	.	T	T	A	A
H17		2		A	T	C	.	.	G	G	.	T	G	.	.	A	G	T	G	T	.	.	.	.	C	T	.	T	T	A	A
H18		1		.	.	.	.	.	.	.	.	.	G	.	.	.	.	.	.	T	.	.	.	.	.	.	.	.	.	.	.

**Table 5:** Haplotype diversity in Sicilian donkeys D-loop sequence (nt 15,386-15,865).

Breed	n	S	SpI	Ss	NH	PH	π ± s.d.	Hd ± s.d.	k
Grigio	26	25	20	5	13	8	0.0220 ± 0.0030	0.871 ± 0.056	8.237
Pantesco	23	1	1	0	2	0	0.0008 ± 0.0003	0.300 ± 0.105	0.300
Ragusano	32	23	21	2	12	5	0.0250 ± 0.0015	0.859 ± 0.048	9.246
Whole sample	82	28	25	3	18	13	0.0218 ± 0.0017	0.812 ± 0.036	7.981

n = sample size; S = total polymorphic sites; SpI = parsimony informative; Ss = singleton site; NH = number of haplotypes; PH = private haplotype; π = nucleotide diversity with their standard deviation (s.d.); Hd: haplotype diversity with their standard deviation (s.d.); k = average number of nucleotide differences (k) within and across the examined populations.

## Data Availability

The raw data supporting the conclusions of this article will be made available by the authors on request.

## Supplementary Materials

The following supporting information can be downloaded at: https://www.mdpi.com/article/10.3390/ani16010090/s1. Supplementary File S1. Table S1. Pairwise genetic differentiation Kst among breeds based on mtDNA.
