# Supplementary material for: Variability Survey at Different Genetic Markers as Effective Tools for the Management of the Endangered Breeds: The Case of the Sicilian Native Donkeys
Source: Animals (Basel). 2025 Dec 28;16(1):90. doi: 10.3390/ani16010090 (PMC12785130; doi:10.3390/ani16010090)
Supplement: Supplementary file 1 [file animals-16-00090-s001.zip › animals-4052043-supplementary.pdf]

Supplementary File S1.

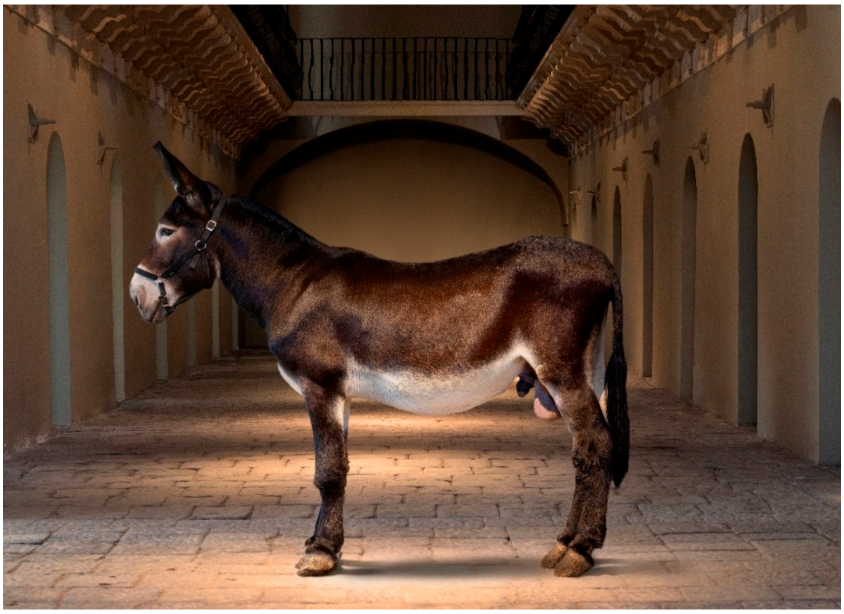

Ragusano

The Ragusano donkey was officially recognized as a breed in 1953. It is an animal with a lively and energetic temperament whose aptitudes range from packing to shooting and milling production. In recent decades, this breed has become the most commonly used in Sicily for the production of milk for use in the human diet and the cosmetics industry.

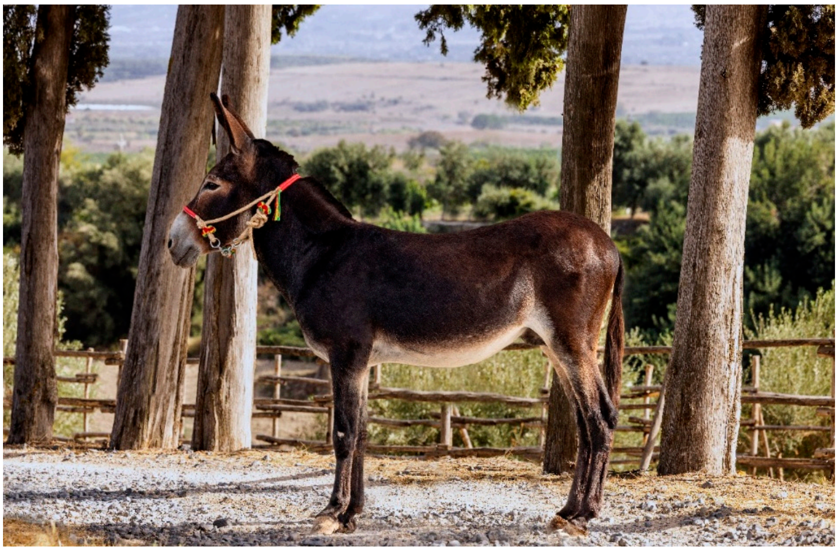

Pantesco

This is an ancient breed originating from the island of Pantelleria; its progenitor of African origins was brought to the island by the Arabs who used it in agriculture. Currently, 100 subjects are recorded in the registry for equine breeds and populations of limited diffusion. They are very robust medium-sized donkeys with a fast and safe gait. The Pantesco donkeys have a great ability to adapt to extreme breeding conditions.

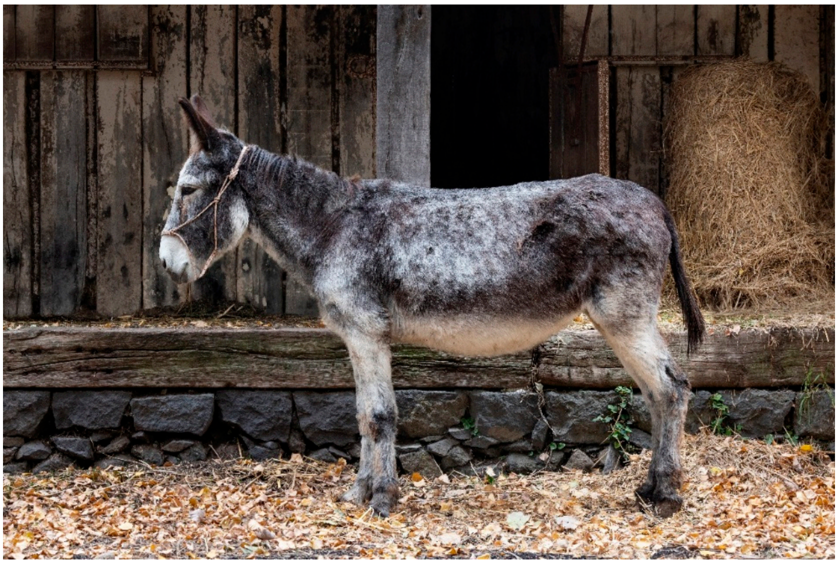

Grigio Siciliano

The origins of the Grigio Siciliano donkey population are very ancient. At the end of the 19th century there was a working donkey, the Siciliano, of small size, with a nonuniform coat but with the lower regions of the body constantly white. Today in Sicily, around 100 subjects have been registered with gray coats, characterized by notable rusticity and frugality. A stud book has not yet been established for this donkey population.

Table S1. *Pairwise genetic differentiation of Kst among breeds based on mtDNA.*

Breed	Kst	P-value
Pantesco vs. Ragusano	0.313	<0.001
Pantesco vs. Grigio	0.133	0.003
Grigio vs. Ragusano	0.049	0.038
